# Machine learning prediction of the failure of high-flow nasal oxygen therapy in patients with acute respiratory failure

**DOI:** 10.1038/s41598-024-52061-z

**Published:** 2024-01-21

**Authors:** Ziwen Wang, Yali Chao, Meng Xu, Wenjing Zhao, Xiaoyi Hu

**Affiliations:** 1 Department of Intensive Care Unit, The Affiliated Hospital of Xuzhou Medical University, Xuzhou, 221000 Jiangsu People’s Republic of China; 2 Department of Anesthesiology, The Second Affiliated Hospital of Nanjing Medical University, Nanjing, 210000 Jiangsu People’s Republic of China

**Keywords:** Diseases, Medical research, Risk factors

## Abstract

Acute respiratory failure (ARF) is a prevalent and serious condition in intensive care unit (ICU), often associated with high mortality rates. High-flow nasal oxygen (HFNO) therapy has gained popularity for treating ARF in recent years. However, there is a limited understanding of the factors that predict HFNO failure in ARF patients. This study aimed to explore early indicators of HFNO failure in ARF patients, utilizing machine learning (ML) algorithms to more accurately pinpoint individuals at elevated risk of HFNO failure. Utilizing ML algorithms, we developed seven predictive models. Their performance was evaluated using various metrics, including the area under the receiver operating characteristic curve, calibration curve, and precision recall curve. The study enrolled 700 patients, with 490 in the training group and 210 in the validation group. The overall HFNO failure rate was 14.1% among the 700 patients. The ML algorithms demonstrated robust performance in our study. This research underscores the potential of ML techniques in creating clinically relevant models for predicting HFNO outcomes in ARF patients. These models could play a pivotal role in enhancing the risk management of HFNO, leading to more patient-centered and personalized care approaches.

## Introduction

Acute respiratory failure (ARF) ranks among the most prevalent conditions in intensive care unit (ICU). The emergence of COVID-19 in Wuhan has sparked a surge in research focused on acute respiratory failure, as the medical community strives to enhance treatments and patient outcomes. Predominantly impacting the respiratory system, COVID-19 has led to a swift progression to ARF in a significant number of patients^1^. Recognized by the World Health Organization as a major international health threat, the pandemic has prompted extensive exploration of various treatment methods^2^. Among these, high-flow nasal oxygen (HFNO) therapy has been noted in several studies as a potentially safe option, even for patients with moderate to severe conditions^3^.

HFNO has emerged as a prominent non-invasive respiratory approach, extensively utilized for treating ARF in recent years^4–6^. This therapy is adept at delivering a consistent and precise fraction of inspired oxygen, enhancing the partial arterial oxygen pressure. Additionally, HFNO offers the advantage of providing heated and humidified gas, which aids in the activation of airway mucus cilia and boosts sputum clearance^7–10^. A key feature of HFNO is its capability to supply high-flow rates that align with a patient's inspiratory flow, creating a positive pressure effect and diminishing anatomic dead space. Compared with conventional oxygen therapies, HFNO has been documented to significantly lower the necessity for invasive mechanical ventilation (IMV)^11^. However, other studies indicate that failure of HFNO may inadvertently delay the initiation of IMV, correlating with heightened mortality rates^12–14^. Given these findings, it becomes critical to promptly identify the potential failure of HFNO in ARF patients. This urgency underscores the need for developing effective strategies to assess and mitigate the risks associated with HFNO failure.

A viable trajectory for the advancement of this strategy encompasses the utilization of machine learning (ML). ML is renowned for its capacity to assimilate and analyze an exceedingly vast array of input variables, culminating in the generation of models with high predictive accuracy^15^. Notably, ML methodologies excel in their ability to delineate and interpret nonlinear correlations and interactions, often surpassing the performance metrics of conventional logistic regression models^16^. To our knowledge, the establishment of such a model, specifically tailored for predicting the failure of HFNO in patients afflicted with ARF, remains an uncharted domain.

Therefore, we embarked on a retrospective data collection of patients diagnosed with ARF who were administered HFNO therapy. We aimed to develop and validate predictive models, employing ML methodologies, that are capable of forecasting the failure of HFNO in treating patients with ARF.

## Methods

### Participants

This was a retrospective study, registered at chictr.org (ChiCTR2300067597). This retrospective study was carried out in the ICU of the Affiliated Hospital of Xuzhou Medical University and was approved by the ethics committee (approved number: XYFY2022-KL464). Due to the retrospective and observational nature of the study, informed consent was waived. The Affiliated Hospital of Xuzhou Medical University, located in Xuzhou City, Jiangsu Province, China, is a tertiary hospital. The medical institution has two hospitals, the east and west hospitals, with 4150 beds.

Patients with ARF who received HFNO were screened for enrollment. Inclusion criteria: (1) diagnosed as ARF (defined as oxygenation index ≤ 300 mmHg, the oxygenation index is the percentage of arterial partial pressure of oxygen divided by the concentration of inspired oxygen) and given HFNO; (2) the age between 18 and 89 years old. Exclusion criteria: (1) ICU stay < 24 h; (2) multiple admissions to ICU; (3) Patients with incomplete clinical data.

### Clinical data characteristics

The characteristics of clinical data in the study are shown in Table 1. The clinical data included: (1) baseline characteristics and comorbidities; (2) vital signs, Glasgow Coma Scale (GCS) score, clinical variables on the first day of HFNO; (3) treatment measures (use of sedation, vasopressors, albumin, diuretics and glucocorticoids); (4) primary outcome. HFNO failure was defined as either application of invasive mechanical ventilation or switching to the other study treatment modality.

**Table 1:** Characteristics of patients in training and testing data set.

Variables	Total (n = 700)	Success group (n = 601)	Failure group (n = 99)	Validation set (n = 210)	Training set (n = 490)	*P*
Age	49.00 (41.00, 60.00)*	49.00 (41.00, 59.00)*	49.00 (41.00, 62.00)*	51.00 (43.00, 60.75)*	49.00 (41.00, 59.75)*	0.085
Gender						0.543
Female	273 (39%)	228 (38%)	45 (45%)	86 (41%)	187 (38)%	
Male	427 (61%)	373 (62%)	54 (55%)	124 (59%)	303 (62%)	
BMI	27.89 (23.97, 32.88)*	27.97 (24.09, 32.87)*	27.27 (22.68, 33.18)*	27.34 (23, 32.65)*	28.17 (24.13, 32.94)*	0.213
Smoking						0.143
No	463 (66%)	391 (65%)	72 (73%)	130 (62%)	333 (68%)	
Yes	237 (34%)	210 (35%)	27 (27%)	80 (38%)	157 (32%)	
SOFA	6.00 (4.00, 9.00)*	6.00 (4.00, 8.00)*	9.00 (7.00, 11.00)*	6.00 (4.00, 9.00)*	6.00 (4.00, 8.00)*	0.279
LODS	6.00 (4.00, 8.00)*	5.00 (3.00, 7.00)*	9.00 (7.00, 11.00)*	6.00 (4.00, 8.00)*	6.00 (4.00, 8.00)*	0.501
SAPSII	41.00 (34.00, 50.00)*	40.00 (33.00, 48.00)*	50.00 (43.50, 62.00)*	41.00 (35.00, 50.75)*	40.50 (33.00, 50.00)*	0.385
GCS	14.00 (9.00, 15.00)*	14.00 (10.00, 15.00)*	9.00 (4.00, 13.00)*	14.00 (9.00, 14.00)*	14.00 (9.25, 15.00)*	0.497
Hypertension						0.276
No	343 (49%)	289 (48%)	54 (55%)	110 (52%)	233 (48%)	
Yes	357 (51%)	312 (52%)	45 (45%)	100 (48%)	257 (52%)	
Diabetes						0.427
No	426 (61%)	368 (61%)	58 (59%)	133 (63%)	293 (60%)	
Yes	274 (39%)	233 (39%)	41 (41%)	77 (37%)	197 (40%)	
Coronary atherosclerotic heart disease						1.000
No	388 (55%)	324 (54%)	64 (65%)	116 (55%)	272 (56%)	
Yes	312 (45%)	277 (46%)	35 (35%)	94 (45%)	218 (44%)	
COPD						0.751
No	676 (97%)	580 (97%)	96 (97%)	204 (97%)	472 (96)%	
Yes	24 (3%)	21 (3%)	3 (3%)	6 (3%)	18 (4%)	
Cerebral infarction						0.441
No	676 (97%)	581 (97%)	95 (96%)	205 (98%)	471 (96%)	
Yes	24 (3%)	20 (3%)	4 (4%)	5 (2%)	19 (4%)	
Peripheral vascular disease						0.338
No	663 (95%)	572 (95%)	91 (92%)	202 (96%)	461 (94%)	
Yes	37 (5%)	29 (5%)	8 (8%)	8 (4%)	29 (6%)	
Urinary tract infections						1.000
No	577 (82%)	498 (83%)	79 (80%)	173 (82%)	404 (82%)	
Yes	123 (18%)	103 (17%)	20 (20%)	37 (18%)	86 (18%)	
Asthma						0.534
No	667 (95%)	572 (95%)	95 (96%)	198 (94%)	469 (96%)	
Yes	33 (5%)	29 (5%)	4 (4%)	12 (6%)	21 (4%)	
AKI						0.105
No	427 (61%)	392 (65%)	35 (35%)	118 (56%)	309 (63%)	
Yes	273 (39%)	209 (35%)	64 (65%)	92 (44%)	181 (37%)	
Sedation use						0.637
No	203 (29%)	170 (28%)	33 (33%)	64 (30%)	139 (28%)	
Yes	497 (71%)	431 (72%)	66 (67%)	146 (70%)	351 (72%)	
Vasopressors use						0.560
No	300 (43%)	251 (42%)	49 (49%)	86 (41%)	214 (44%)	
Yes	400 (57%)	350 (58%)	50 (51%)	124 (59%)	276 (56%)	
Diuretics use						0.509
No	52 (7%)	44 (7%)	8 (8%)	13 (6%)	39 (8%)	
Yes	648 (93%)	557 (93%)	91 (92%)	197 (94%)	451 (92%)	
Albumin use						0.437
No	509 (73%)	425 (71%)	84 (85%)	148 (70%)	361 (74%)	
Yes	191 (27%)	176 (29%)	15 (15%)	62 (30%)	129 (26%)	
Glucocorticoids use						1.000
No	637 (91%)	550 (92%)	87 (88%)	191 (91%)	446 (91%)	
Yes	63 (9%)	51 (8%)	12 (12%)	19 (9%)	44 (9%)	
Prone position						0.402
No	616 (88%)	520 (87%)	96 (97%)	181 (86%)	435 (89%)	
Yes	84 (12%)	81 (13%)	3 (3%)	29 (14%)	55 (11%)	
Heart_rate	85.34 (76.11, 95.75)*	84.76 (76.33, 93.59)*	90.52 (73.08, 105.05)*	85.08 (77.14, 96.98)*	85.45 (75.78, 95.60)*	0.873
Temperature	36.71 (36.49, 37.06)*	36.72 (36.49, 37.05)*	36.69 (36.49, 37.17)*	36.73 (36.51, 37.08)*	36.71 (36.49, 37.05)*	0.639
Mean blood pressure	73.42 (68.43, 79.06)*	73.67 (68.67, 78.92)*	72.35 (66.08, 79.97)*	73.15 (67.74, 79.15)*	73.56 (68.72, 78.94)*	0.466
Respiratory rate	18.84 (16.74, 21.64)*	18.64 (16.52, 21.34)*	20.71 (17.73, 24.41)*	18.76 (16.65, 21.53)*	18.87 (16.78, 21.69)*	0.835
WBC	12.00 (8.20, 16.10)*	11.80 (8.20, 15.70)*	13.20 (8.65, 18.15)*	12.35 (8.20, 16.92)*	11.85 (8.30, 15.57)*	0.630
Hb	10.05 (8.80, 11.60)*	10.00 (8.80, 11.50)*	10.30 (9.05, 12.20)*	10.15 (8.90, 11.60)*	10.00 (8.80, 11.67)*	0.779
PLT	179.50 (126.00, 235.00)*	180.00 (128.00, 233.00)*	172.00 (118.00, 252.00)*	179.00 (125.25, 239.25)*	180.00 (126.00, 233.00)*	0.973
ALT	42.00 (31.00, 63.00)*	42.00 (32.00, 62.00)*	49.00 (25.50, 83.00)*	42.00 (31.00, 57.00)*	42.00 (32.00, 64.75)*	0.229
AST	30.00 (20.75, 46.00)*	30.00 (21.00, 46.00)*	38.00 (21.00, 55.50)*	34.50 (22.00, 50.00)*	30.00 (20.00, 46.00)*	0.243
ALB	3.20 (3.00, 3.50)*	3.20 (3.00, 3.60)*	3.20 (3.00, 3.45)*	3.30 (3.00, 3.60)*	3.20 (3.00, 3.50)*	0.084
Glycemia	125.75 (96.00, 164.00)*	125.00 (96.00, 161.00)*	134.00 (95.50, 177.25)*	128.00 (101.25, 172.62)*	124.00 (94.00, 161.00)*	0.100
Cr	17.00 (2.28, 31.25)*	16.00 (2.20, 30.00)*	24.00 (2.55, 41.00)*	18.00 (2.12, 33.00)*	16.00 (2.40, 30.00)*	0.240
BUN	1.60 (0.90, 14.00)*	1.50 (0.90, 13.00)*	2.40 (1.25, 22.50)*	1.65 (0.90, 14.00)*	1.50 (0.90, 13.75)*	0.744
Lactic acid	3.72 (3.15, 4.10)*	3.65 (3.15, 4.05)*	3.90 (3.45, 4.25)*	3.80 (3.20, 4.20)*	3.65 (3.15, 4.10)*	0.160
PT	15.50 (13.60, 18.70)*	15.40 (13.60, 18.20)*	16.20 (13.80, 23.10)*	15.15 (13.40, 18.17)*	15.60 (13.70, 18.90)*	0.111
INR	1.40 (1.20, 1.70)*	1.40 (1.20, 1.70)*	1.50 (1.20, 2.20)*	1.40 (1.20, 1.70)*	1.40 (1.20, 1.80)*	0.195
K^+^	4.20 (3.80, 4.70)*	4.20 (3.80, 4.70)*	4.20 (3.85, 4.80)*	4.20 (3.80, 4.60)*	4.20 (3.80, 4.70)*	0.578
Na^+^	139.00 (136.00, 141.00)*	139.00 (137.00, 141.00)*	138.00 (134.50, 141.00)*	139.00 (136.00, 141.00)*	139.00 (136.00, 141.00)*	0.656
Ca^+^	8.30 (7.80, 8.70)*	8.30 (7.90, 8.70)*	8.20 (7.65, 8.70)*	8.30 (7.80, 8.70)*	8.30 (7.80, 8.70)*	0.871
Oxygenation index	151.00 (135.00, 207.00 )*	154 .00 (139.00, 214.00 )*	133.00 (106.00, 149.75)*	151.00 (134.00, 202.12)*	151.00 (136.00, 207.88)*	0.736
Respiratory failure severity						0.466
Mild	193 (28%)	188 (31%)	5 (5%)	54 (26%)	139 (28%)	
Moderate	495 (71%)	407 (68%)	88 (89%)	154 (73%)	341 (70%)	
Severe	12 (2%)	6 (1%)	6 (6%)	2 (1%)	10 (2%)	

*SOFA* Sepsis-related Organ Failure Assessment, *LODS* Logistic Organ Dysfunction Score, *SAPSII* Simplified Acute Physiology Score, *GCS* Glasgow Coma Score, *COPD* chronic obstructive pulmonary disease, *AKI* acute kidney injury, *WBC* white blood cell, *Hb* hemoglobin, *PLT* platelet count, *ALT* alanine aminotransferase, *AST* aspartate aminotransferase, *ALB* albumin, *Cr* creatinine, *BUN* blood urea nitrogen, *PT* prothrombin time, *INR* international normalized ratio; *Median (Q1, Q3).

### Development of machine learning models

The outcome-related feature screening process was carried out using least absolute shrinkage and selection operator (LASSO). For the development of the models, the most relevant features chosen are employed. Non-zero characteristic indicators selected through LASSO analysis were put into the multivariate logistic regression analysis to identify the independent risk factors associated with HFNO failure.

We considered seven different types of models: support vector machine (SVM), adaptive boosting (ADABOOST), logistic regression (LR), extreme gradient boosting (XGBOOST), stacking ensemble algorithms (STACK), random forest (RF), and naive bayes (NB); The STACK is algorithms that integrate LR, SVM, NB, and RF. For the selection of hyper-parameters in models, we used five fold cross-validation for the selection of hyper-parameters, which also helped to effectively prevent the model's over-fitting.

### Model validation

For validation of prediction model, we divided the data randomly into a training set and validation set according to a 70–30 split, and then used the resampling method for the internal validation of the prediction model in training set. Finally, we performed the validation again in the validation set. We provide additional technical information on the methods and parameter settings in the Supplementary material Table 1.

### Model performance and explainability

To evaluate our models, we considered three predictive metrics: area under receiver operating characteristic (AUROC) curve, Brier score and area under precision recall curve (AUPRC). AUROC is bounded between 0.5 and 1.0, with higher values being better. The Brier score is the mean squared difference between the predicted probability of HFNO failure and the actual outcome (0 or 1 where 1 indicates failure of HFNO). The Brier score is bounded between 0 and 1, with lower values being better. We additionally compared the models by plotting their receiver operator characteristic (ROC) curves, precision recall (PR) curves and calibration curve.

We applied the Shapley (SHAP) value to explain features in the training set. The SHAP summary, combining feature importance with feature effects, was visualized with dot plots to present the distribution of SHAP The position on the y-axis was determined by the feature and that on the x-axis by the SHAP value. The features are ranked by importance. Moreover, partial dependence plots (PDPs) were created to visualize the average change in probability of HFNO failure for all values of a predictor while keeping all other predictors constant^17^.

### Sample size and statistical analysis

Pmsampsize package (https://search.r-project.org/CRAN/refmans/pmsampsize/html/pmsampsize.html) in R software computes the minimum sample size required for the development of a new multivariable prediction model using the criteria proposed by Riley et al.^18^. Riley et al. lay out a series of criteria the sample size should meet. These aim to minimize the over-fitting and to ensure precise estimation of key parameters in the prediction model. Following the parameters set in the pmsampsize package, we set the c-statistic to 0.80, the potential prediction parameter to 8, and the target event incidence to be 14.1%. Minimum sample size required for new model development based on the above parameters inputs was 459, with 65 events. The sample size in the training set satisfies the minimum sample size requirement for the development of a new multivariable prediction model.

The Kolmogorov–Smirnov test was used to test the normal distribution for measurement data. Normally distributed data were expressed as means ± standard deviation, and the skewed distributed data was reported as medians with interquartile (25th–75th) percentiles. The two groups were compared using student *t*-test or Mann–Whitney *U* tests. Numeric data were expressed as a percentage (%), using χ^2^ or Fisher’s exact probability tests. R software was used for all analyses (R Foundation for Statistical Computing, Vienna, Austria).

### Ethics statement

The study was approved by the Ethics Committee of the Affiliated Hospital of Xuzhou Medical University (approved number: XYFY2022-KL464).The procedures were followed in accordance with the ethical standards of the Ethics Committee of the Affiliated Hospital of Xuzhou Medical University on human experimentation and with the Helsinki Declaration of 1975. Due to the retrospective and observational nature of the study, informed consent was waived by the Ethics Committee of the Affiliated Hospital of Xuzhou Medical University.

## Results

### Characteristics of participants

During the study period, 1671 patients diagnosed with ARF were initially enrolled. Following the exclusion of 971 patients for various reasons, as detailed in Supplementary Material Fig. 1, the study proceeded with an analysis of 700 patients. These patients were divided into two groups: a training set comprising 490 patients and a validation set consisting of 210 cases. The general characteristics of these groups are summarized in Table 1. There are no statistically significant differences between the training set and the validation set (all *P* > 0.05). In both the training and validation sets, HFNO was failure in 67 (13.7%) of the 490 patients and 32 (15.2%) of the 210 patients, respectively. Overall, the incidence of HFNO failure across the entire dataset was 14.1%. Considering the severity based on the oxygenation index, the majority of patients in this study exhibited mild or moderate symptoms^3^, as detailed in Table 1 and Supplementary Material Fig. 2.

**Figure 1:**
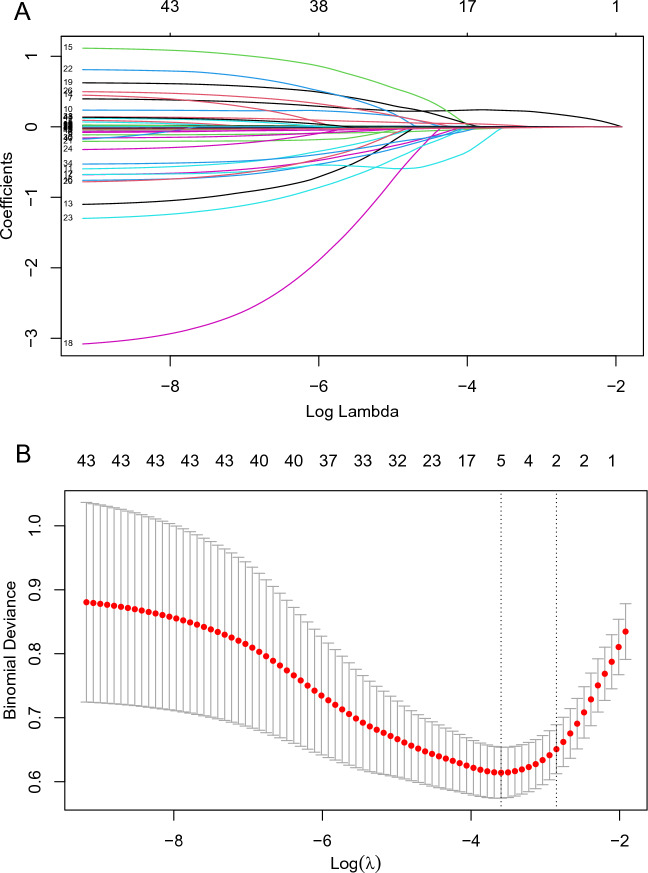
Demographic and clinical feature selection using the LASSO regression. (**A**) The selection of the tuning parameter (lambda) in the LASSO model used fivefold cross-validation with the minimum criteria. The relationship curve between partial likelihood deviation (binomial deviation) and log (lambda) was plotted. Dotted vertical lines were drawn at the optimal values by using the minimum criteria and the 1 standard error (SE) of the minimum criteria (the 1-SE criteria). (**B**) LASSO coefficient profiles of the 44 features. A coefficient profile plot was produced against the log (lambda) sequence. Vertical line was drawn at the value selected using fivefold cross-validation, where optimal lambda resulted in 5 features with non-zero coefficients. *LASSO* least absolute shrinkage and selection operator.

**Figure 2:**
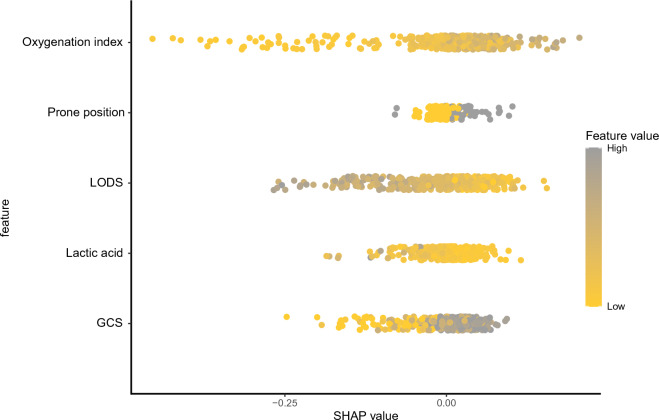
SHAP importance plots of the HFNO failure for the ML model. The position on the y-axis was determined by the feature and that on the x-axis by the SHAP value. The length of the SHAP value indicates the importance of the features. *LODS* Logistic Organ Dysfunction Score, *GCS* Glasgow Coma Score; SHAP, Shapley.

### Feature importance

Table 2 shows the top 5 most important variables of the LASSO regression model for training set. Forty-three variables from the clinical characteristics were included in the LASSO regression analysis (Fig. 1A,B). We selected five non-zero characteristic variables including logistic organ dysfunction score (LODS), Glasgow coma score (GCS), prone position, lactic acid, oxygenation index to construct models (Table 2). We plotted the SHAP importance plots to reflect the significance of the five features. Each row represents the impact of a feature on the outcome of HFNO failure, with higher SHAP values indicating higher likelihood of HFNO failure (see Fig. 2 for details). The PDPs in Supplementary material Fig. 3 shows that an oxygenation index under 155 or lactic acid above 3.5 compared to their median values increases the probability of HFNO failure.

**Table 2:** LASSO regression results of important variables related to HFNOT failure (training dataset).

Variables	Coefficient	Lambda.min
LODS	0.235	0.027
GCS	− 0.021	
Prone position	− 0.053	
Lactic	0.030	
Oxygenation index	− 0.016

**Figure 3:**
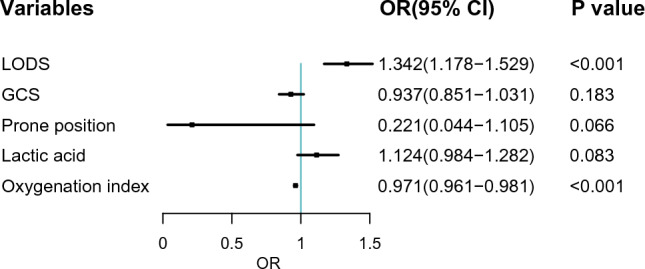
Forest plot of multivariate logistic regression analysis. *LODS* Logistic Organ Dysfunction Score, *GCS* Glasgow Coma Score, *SHAP* Shapley, *OR* odds ratio, *CI* confidence interval.

The results of multivariate logistic analysis are presented in Fig. 3. Independent predictors identified by multivariate logistic regression analysis included LODS (OR = 1.342; 95% CI 1.178–1.529) and oxygenation index (OR = 0.971; 95% CI 0.961–0.981).

### Model performance

**Figure 4:**
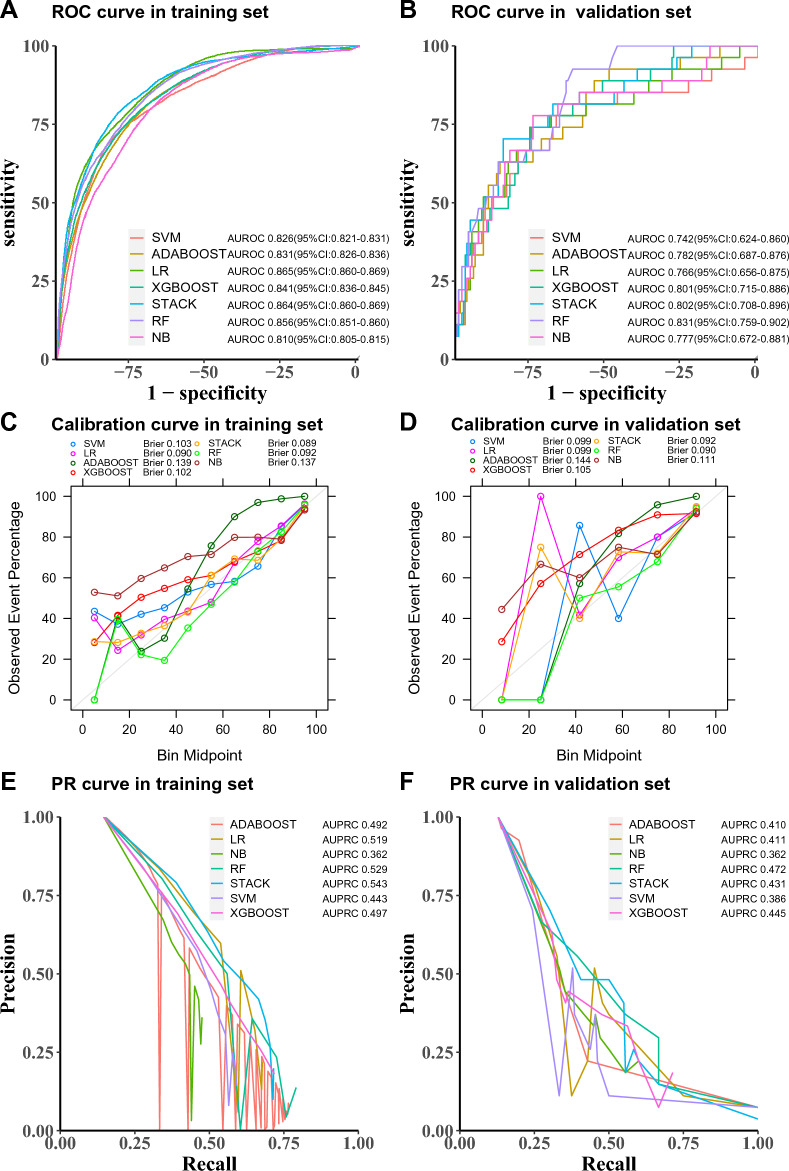
A series of performance metrics in the ML models. (**A**,**B**) The receiver operating characteristic (ROC) curve was compared between training and validation set. (**C**,**D**) The calibration curve in training and validation set. (**E**,**F**) The Precision Recall (PR) curve in training and validation set. *LODS* Logistic Organ Dysfunction Score, *GCS* Glasgow Coma Score, *SHAP* Shapley, *OR* odds ratio, *CI* confidence interval, *AUROC* area under receiver operating characteristic, *AUPRC* area under precision-recall curve.

Figure 4 displays the AUROC, Brier score and AUPRC metrics for the different predictive models and using different sets of data. In the training set, all models resulted in AUROC on the order of 0.81 to 0.87. There were no statistically significant differences between the AUROC for all models through the DeLong's test (*P* > 0.05). However, only three models in the validation set had AUROC greater than 0.80. Specifically, the RF model's AUROC showed the least difference in the training and validation. To further compare the models, we additionally compared the models by plotting calibration curves and their PR curves. The STACK and RF models have lower Brier scores, and their calibration curves also have higher agreement with the 45-degree line. Similarly, the RF model's Brier score showed the least difference in the training and validation sets. The larger AUPRC represents the better performance of the model. In training set, only three models reached AUPRC above 0.5, which are LR, RF and STACK models respectively. However, in the validation set, RF has a larger AUPRC. Similarly, the RF model's AUPRC showed the least difference in the training and validation sets. In view of the above results analysis, the RF model is deemed superior to the other models.

Finally, we established a dynamic grading system to facilitate the application of the model. The website address of the dynamic scoring system is https://huxiaoyi.shinyapps.io/whole/.

## Discussion

ARF remains a leading cause of mortality among patients in ICU. Recently, HFNO has gained prominence in the treatment of ARF, effectively reducing the necessity for IMV^19,20^. However, the failure of HFNO therapy can lead to prolonged ICU stays and increased mortality rates^21^. Therefore, early prediction of HFNO failure is crucial for identifying patients at higher risk and optimizing their treatment strategies.

Since the COVID-19 outbreak in 2020, the European Society for Critical Care has released clinical practice guidelines for HFNO^22^. In 2021, several Chinese medical associations also issued expert consensus guidelines on HFNO's clinical use^23^. These guidelines emphasize close monitoring of patients’ vital signs within the first 1–2 h of HFNO application. They recommend upgrading respiratory support if failure predictors are observed, including a respiratory rate over 35 breaths/min, SpO_2_ below 88%, a ROX index under 2.85, contradictory thoracic and abdominal movements, or the use of accessory respiratory muscles. Although these guidelines are based on moderate-level evidence, there remains a gap in research to enhance this evidence level. The relatively recent introduction of HFNO as a treatment limits the availability of extensive data. This study aims to contribute valuable insights for monitoring HFNO, addressing this data scarcity.

Our data analysis reveals two crucial insights regarding the prediction of HFNO failure in ARF patients. Firstly, all models exhibit high discrimination in the training set, with some achieving an AUROC between 0.80 and 0.85. Secondly, the ability of these models to resist over-fitting, despite the inclusion of numerous features, is key to our methodology's effectiveness^24^. Traditional risk model development often follows the “one-in-ten” rule to limit features and prevent over-fitting, a constraint primarily due to the limitations of classic logistic regression. This traditional approach requires significant manual intervention and expert knowledge to exclude unnecessary features. ML algorithms can be helpful in developing more precise prognostication models that integrate complex interactions at a higher dimensional level^25^. Physicians now have access to a variety of resources to learn about ML fundamentals and techniques^26,27^. In our study's training set, the classical logistic regression model showed a higher AUROC, but it also exhibited the largest drop in the validation set, with a 0.099 AUROC difference, suggesting potential over-fitting. Our findings further confirm that ML models are generally more robust than traditional logistic regression models. However, despite their advanced algorithmic power, ML models, except LR, are often “black-box” algorithms, offering high algorithmic capabilities but low interpretability^28^. This raises several concerns: (1) Clinicians may find it challenging to explain ML-based decisions, hindering the adoption of ML for critical decisions, and (2) Emerging regulations and concerns about ML emphasize the need for interpretability and transparent predictive reasoning. To address these issues, our study includes forest plots of multivariate logistic analysis and SHAP importance plots to better elucidate the models' characteristics.

In our study, we analyzed a total of 700 patients, among whom 99 cases (14.1%) experienced HFNO failure, as detailed in Table 1. The low failure rate of HFNO in our study can be attributed to four primary factors: (1) The patients with respiratory failure included in our study predominantly had mild or moderate symptoms, evidenced by a median oxygenation index of 151.00. (2) The patient cohort was relatively young, with a median age of 49 years. (3) The retrospective nature of the study introduces inherent biases. (4) The use of HFNO was often complemented by the application of prone positioning. Therefore, the success rate of HFNO observed in our study surpasses that reported in previous research^29,30^.

The independent risk factors related to HFNO failure including LODS and oxygenation index were identified (Fig. 3). Among these features, oxygenation index was the strongest factor for HFNO failure in patients with ARF, and its SHAP value is the higher among several features (Fig. 2). Therefore, patients with lower oxygenation index also had a higher risk of failure in HFNO. Previous studies on the prognosis of pulmonary infection induced sepsis showed that oxygenation index was an independent risk factor for predicting in-hospital mortality^31,32^. The results of Liu et al.^33^ also confirmed that oxygenation index was an independent risk factor for patients with non-invasive ventilation failure. LODS is an organ function-focused scoring system that reflects the severity of multiple organ dysfunction syndrome (MODS)^34^. This study also found that LODS was a independent risk factor for the failure of HFNO. In addition to the oxygenation index, it is also an important feature in ML models (see Fig. 3 for details). Finally, the ML model was transformed into a dynamic scoring system, which further facilitated the use of this model and patient’s understanding of disease prognosis.

In this study, there were several limitations that are inherent in these types of retrospective, ML projects. First of all, this study uses retrospective data, and should continue to conduct prospective validation research. Secondly, external validation data from other institutions can further determine the extrapolation of this model. Thirdly, although the ultimately validated ML model was robust and accurate, the size of data used was still relatively small. Fourthly, variable selection was exclusively conducted using the LASSO method. We did not employ other variable selection algorithms like RF, Boruta, etc., which could potentially have further enhanced the model's performance. Fifth, patients who had multiple ICU admissions were excluded, and only those aged between 18 and 89 years were included. The reasons for this are as follows: (1) To avoid the impact of duplicate data; (2) To maintain the independence of the dataset; (3) To reduce the potential for confounding factors; (4) Children or patients older than 90 years are difficult to cooperate with high-flow nasal catheter oxygen therapy. Their poor compliance with HFNO could potentially bias the outcomes. Finally, many features associated with HFNO failure are complex and there are far more factors to be investigated and used to predict the failure of HFNO. Thus, picture features such as chest X-ray computer tomography should be included to improve the model in the future.

## Conclusion

In this study, this work demonstrates the ability of ML techniques to produce clinically useful models for predicting state of HFNO.

The study may assist risk management of HFNO with improved patient centered and personalized care.

### Supplementary Information

Supplementary Information.

## Data Availability

The datasets used and analysed in this study may be obtained from the corresponding author upon reasonable request.

## Abbreviations

ARF: Acute respiratory failure
HFNO: High-flow nasal oxygen
ML: Machine learning
LASSO: Least absolute shrinkage and selection operator
IMV: Invasive mechanical ventilation
AUROC: Area under the receiver operating characteristic
AUPRC: Area under precision recall curve
PR: Precision recall
SHAP: Shapley
PDPs: Partial dependence plots
SOFA: Sepsis-related organ failure assessment
LODS: Logistic organ dysfunction score
SAPSII: Simplified acute physiology score
GCS: Glasgow coma score
COPD: Chronic obstructive pulmonary disease
AKI: Acute kidney injury
WBC: White blood cell
Hb: Hemoglobin
PLT: Platelet count
ALT: Alanine aminotransferase
AST: Aspartate aminotransferase
ALB: Albumin
Cr: Creatinine
BUN: Blood urea nitrogen
PT: Prothrombin time
INR: International normalized ratio
SVM: Support vector machine
ADABOOST: Adaptive boosting
LR: Logistic regression
XGBOOST: Extreme gradient boosting
STACK: Stacking ensemble algorithms
RF: Random forest
NB: Naive bayes
