# Supplementary material for: Machine learning prediction of the failure of high-flow nasal oxygen therapy in patients with acute respiratory failure
Source: Sci Rep. 2024 Jan 21;14:1825. doi: 10.1038/s41598-024-52061-z (PMC10800339; doi:10.1038/s41598-024-52061-z)
Supplement: Supplementary file 1 — Supplementary Information. [file 41598_2024_52061_MOESM1_ESM.docx]

**Supplementary material Table 1. Tuning parameters in five fold cross validation for each algorithm**

| Test Model name | Tuning Parameters |
| --- | --- |
| SVM | sigma=0.0237320232898012, C=0.50404922424343, method=svmRadial |
| ADABOOST | mfinal=43, maxdepth=3, coeflearn=Zhu, method=AdaBoost.M1 |
| LR | None |
| XGBOOST | nrounds=291, max_depth=4, eta=0.51928486479912, gamma=0.526228714734316, colsample_bytree=0.472246981598437, min_child_weight=3, subsample=0.998413218942005, method=xgbTree |
| STACK | None |
| RF | mtry=1, method=rf |
| NB | laplace=0, usekernel=FALSE, adjust=1, method=naive_bayes |

多大

SVM，Support Vector Machine; ADABOOST, Adaptive Boosting; LR, logistic regression; XGBOOST, Extreme Gradient Boosting; STACK, Stacking Ensemble;RF, Random Forest; NB, Naive Bayes;


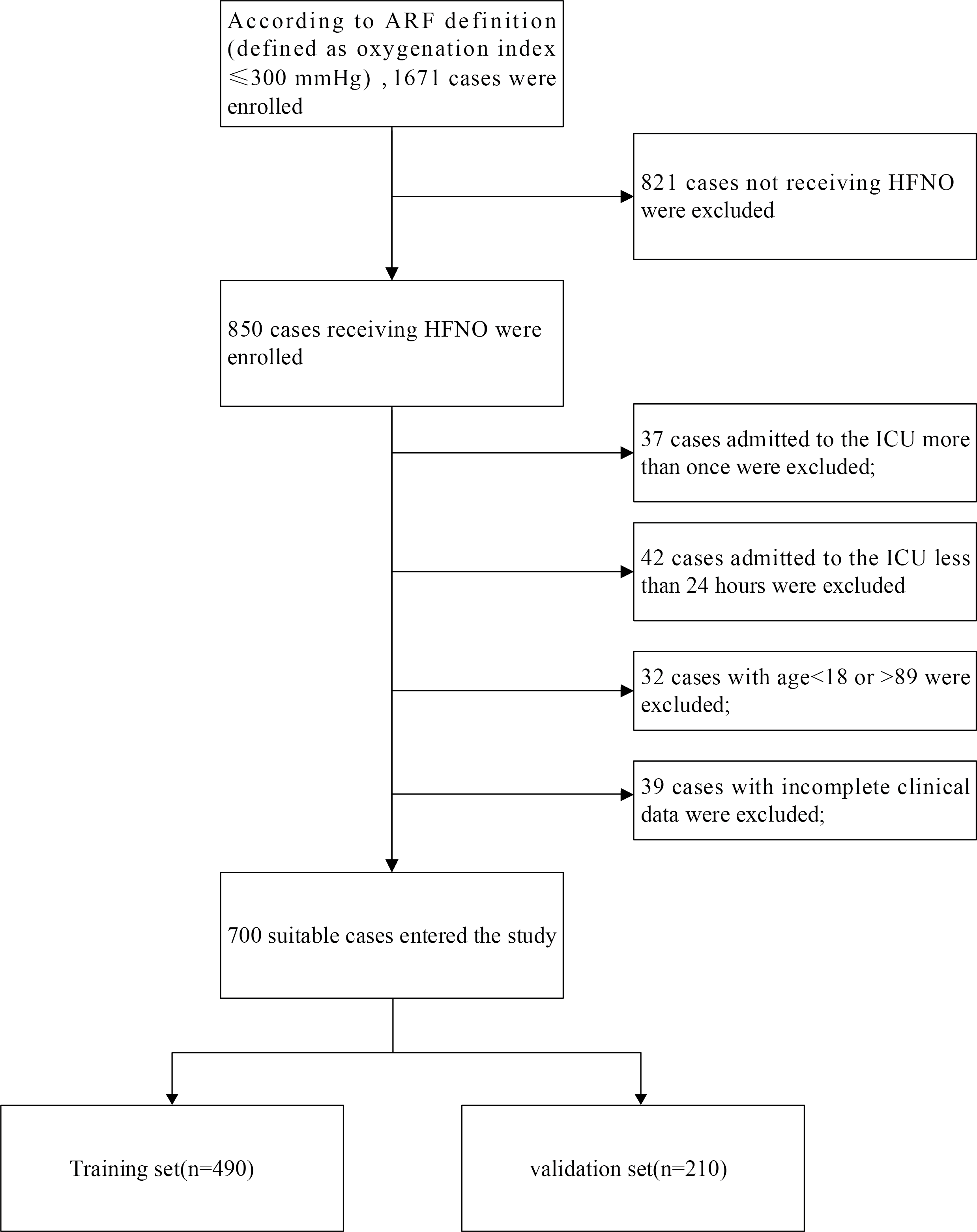


# Supplementary material Figure 1. Flowchart.

# Flow chart of patient recruitment.
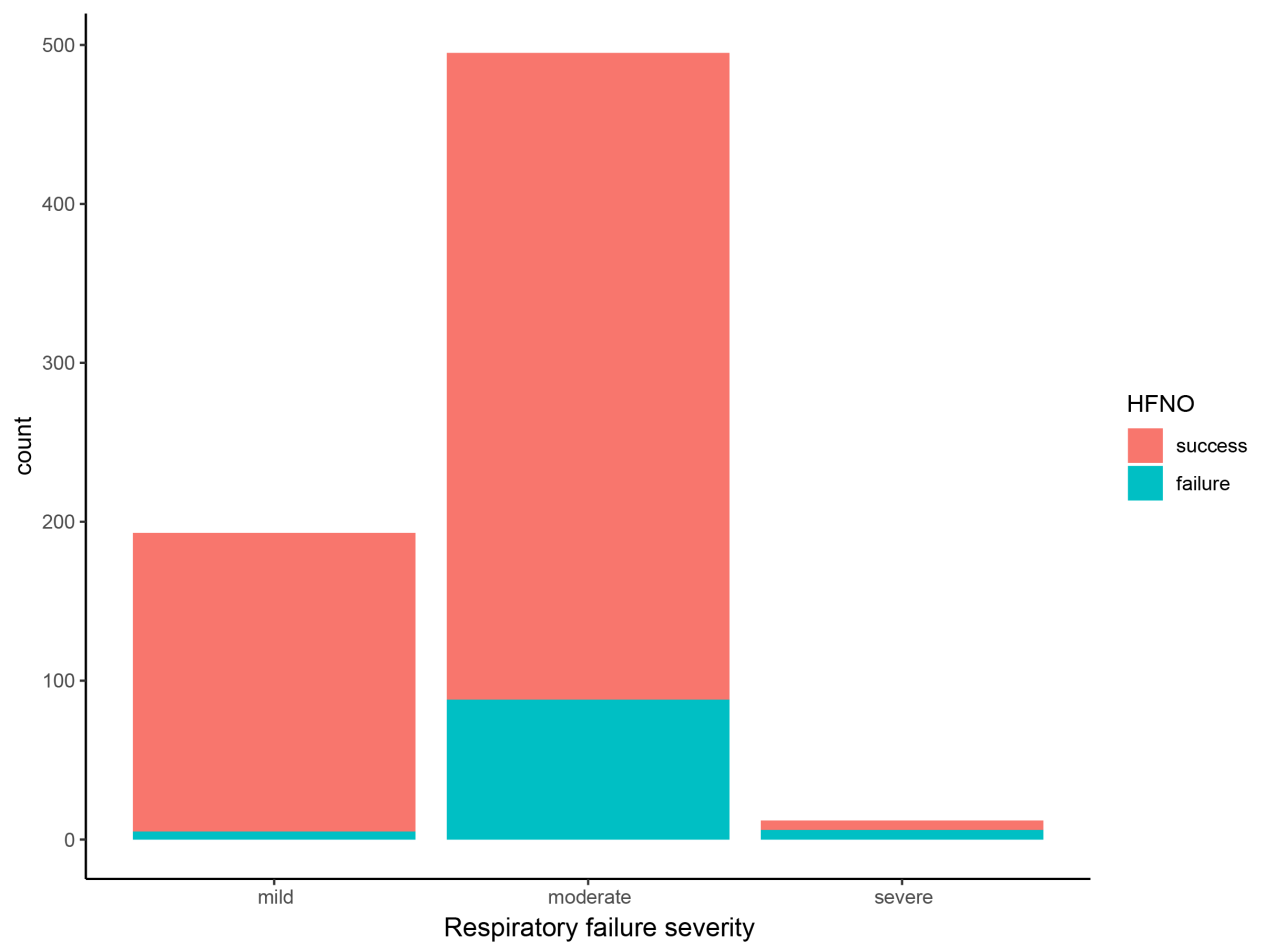


**Supplementary material Figure 2. Respiratory failure severity.**

Distribution of success and failure in high-flow oxygen therapy in patients with different respiratory failure severity. HFNO, High-Flow Nasal Oxygen; All data are presented as count;


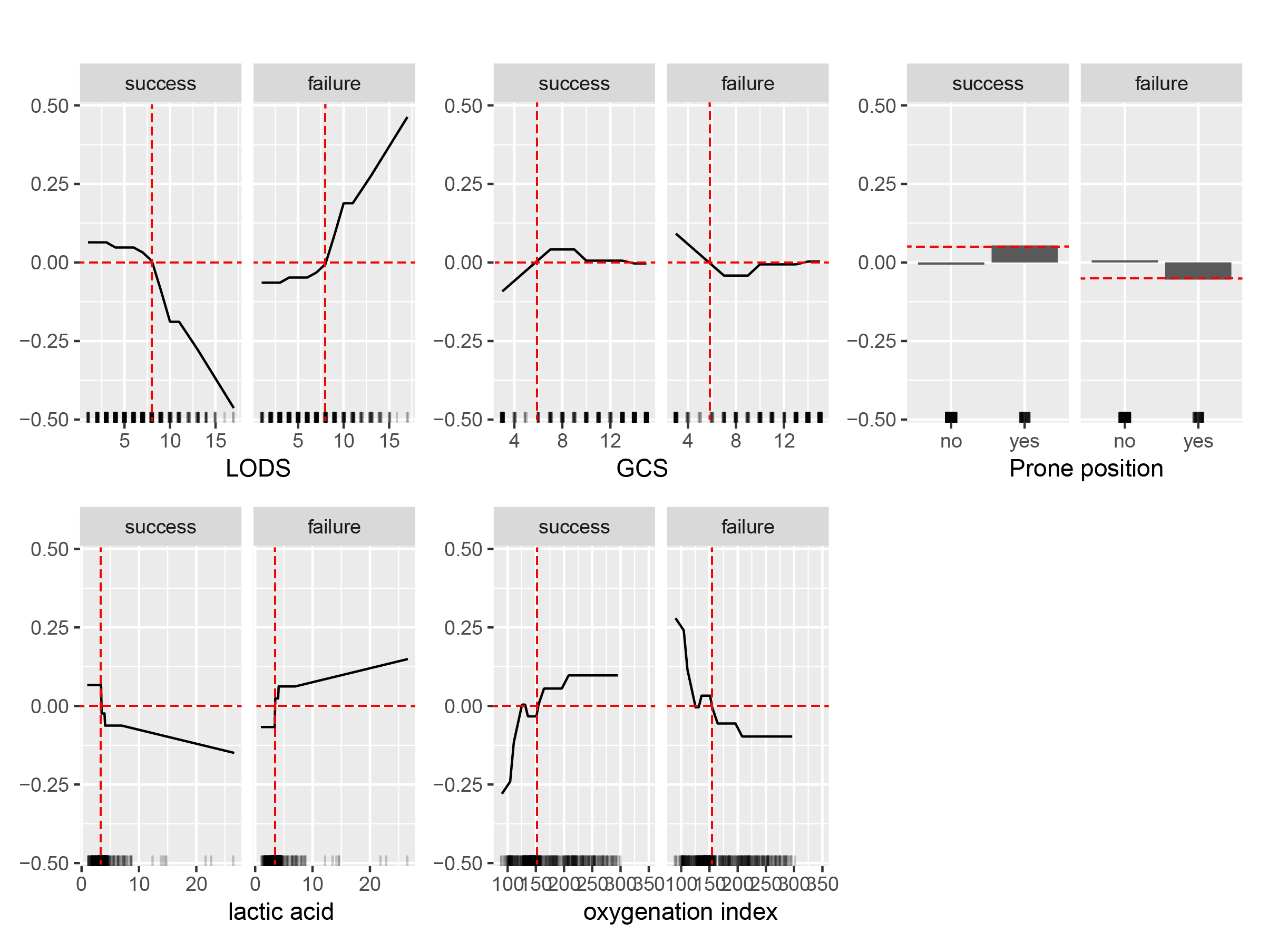


**Supplementary material Figure 3. Partial dependence plots.**

The best truncated distribution of the different variables in the included model. The red line represents that the variable begins to have the opposite effect on the outcome after the variable reaches the cutoff value in the model.
